# Salicylic acid carboxyl glucosyltransferase UGT87E7 regulates disease resistance in *Camellia sinensis*

**DOI:** 10.1093/plphys/kiab569

**Published:** 2021-12-06

**Authors:** Yunqing Hu, Mengting Zhang, Mengqian Lu, Yi Wu, Tingting Jing, Mingyue Zhao, Yifan Zhao, Yingying Feng, Jingming Wang, Ting Gao, Zixiang Zhou, Bin Wu, Hao Jiang, Xiaochun Wan, Wilfried Schwab, Chuankui Song

**Affiliations:** 1 State Key Laboratory of Tea Plant Biology and Utilization, International Joint Laboratory on Tea Chemistry and Health Effects, Anhui Agricultural University, 230036 Hefei, Anhui, China; 2 Biotechnology of Natural Products, Technische Universität München, Liesel-Beckmann-Str. 1, 85354 Freising, Germany

## Abstract

Plant immune response following pathogenic infection is regulated by plant hormones, and salicylic acid (SA) and its sugar conjugates play important roles in establishing basal resistance. Here, the important pathogen *Pseudopestalotiopsis camelliae-sinensis* (*Pcs*) was isolated from tea gray blight, one of the most destructive diseases in tea plantations. Transcriptomic analysis led to the discovery of the putative *Camellia sinensis* UDP-glucosyltransferase *CsUGT87E7* whose expression was significantly induced by SA application and *Pcs* infection. Recombinant *Cs*UGT87E7 glucosylates SA with a K_m_ value of 12 µM to form SA glucose ester (SGE). Downregulation reduced the accumulation of SGE, and *CsUGT87E7*-silenced tea plants exhibited greater susceptibility to pathogen infection than control plants. Similarly, *CsUGT87E7*-silenced tea leaves accumulated significantly less SA after infection and showed reduced expression of pathogenesis-related genes. These results suggest that *Cs*UGT87E7 is an SA carboxyl glucosyltransferase that plays a positive role in plant disease resistance by modulating SA homeostasis through a mechanism distinct from that described in Arabidopsis (*Arabidopsis thaliana*). This study provides insight into the mechanisms of SA metabolism and highlights the role of SGE in the modulation of plant disease resistance.

## Teaser

The glucosyltransferase UGT87E7 from *Camellia sinensis* specifically forms a salicylic acid glucose ester and plays a positive role in plant disease resistance by modulating salicylic acid homeostasis.

## Introduction

Plants face various environmental pressures while growing in nature, including biotic and abiotic stresses. Over the long course of plant adaptation to the environment, plants have acquired a multistage immune system to protect themselves against pathogens. Plant immune response includes pathogen-associated molecular pattern-triggered immunity, which allows plants to fend off a large number of potential pathogens (Li et al., 2005), and effector-triggered immunity (Jones and Dangl, 2006), which relies on proteins encoded by resistance (R) genes to induce a stronger and more durable immune response (Chisholm et al., 2006). When plants are exposed to pathogens, some responses are limited to the infested damaged organ, while other responses systemically spread far from the infested organ and affect the whole plant (Conrath et al., 2015; Vlot et al., 2021). These latter responses include systemic-acquired resistance (SAR) and induced systemic resistance (ISR), depending on the site of induction and the lifestyle of the pathogenic microorganism (Choudhary et al., 2007; Vlot et al., 2021). Small metabolites, such as salicylic acid (SA; Lim et al.,2020), glycerol-3-phosphate (Chanda et al., 2011), azelaic acid (Jung et al., 2009), dehydroabietinal (Chaturvedi et al., 2008), pipecolic acid (Pip; Návarová et al., 2012) and *N*-hydroxy-pipecolic acid (NHP; Chen et al., 2018; Hartmann et al., 2018) have been reported to be involved in long-distance communication and plant immunity (Shah et al., 2014; Vlot et al., 2021). Among them, SA plays a vital role in the activation of defense mechanisms during pathogen infection (Vlot et al., 2009) and is associated with the accumulation of pathogenesis-related (PR) proteins (Ross, 1961; Durrant and Dong, 2004).

The plant hormone SA plays a regulatory role in many physiological and biochemical processes, serves as a critical signal for the activation of disease resistance in plants, and participates in plant immune response (Vlot et al., 2009; Ding and Ding, 2020). Once synthesized, free SA may undergo several biologically relevant chemical modifications, including glucosylation and methylation. Most modifications render SA inactive, permitting the fine-tuning of its accumulation, function, and/or mobility (Dempsey et al., 2011). In plants, small hydrophilic molecules such as glucose are often conjugated to SA to aid in transport and storage because of their reactivity and hydrophilicity (Thompson et al., 2017). MeSA, the methylated derivative of SA, has been reported to act as a signaling compound in SAR in *Nicotiana benthamiana* (Park et al., 2007). A different study, however, indicated that neither MeSA nor JA is essential for systemic immunity, emphasizing the crucial role of SA in Arabidopsis (*Arabidopsis thaliana*) (Attaran et al., 2009). SA glucose exists in two forms, SA 2-*O*-β-d-glucoside (SAG) and SA glucose ester (SGE). In Arabidopsis, in vitro catalytic analyses determined that the glycosyltransferase UGT74F1 can form SAG, whereas UGT74F2 produces both SAG and SGE (Lim et al., 2002). The *A.* *thaliana* glucosyltransferase UGT76B1-mediated glucosylation controls the levels of active SA, NHP, and isoleucic acid in concert to balance plant defense and growth (Bauer et al., 2021; Cai et al., 2021; Hõrak, 2021; Mohnike et al., 2021, Zeier, 2021). SAG is thought to be a more stable storage form for small phenolics, and its levels have been shown to increase in parallel with free SA in plant defense responses, suggesting that it is associated with plant defense (Enyedi et al., 1992; Chen et al., 1995). SGE is thought to be a high-energy compound and biosynthetic intermediate (Bowles et al., 2006), but the formation of SGE and its role(s) in plant defense is still not understood (Boachon et al., 2014).

Tea is considered to be the most popular nonalcoholic beverage and is produced from leaves of the tea plant (*Camellia sinensis*) (Zhao et al., 2020). With the continuous expansion of tea plantation area globally, damage from tea diseases has seriously affected tea production and quality. The leaf disease, tea gray blight, also known as tea shoot wilt, is one of the main diseases of tea plants in China (Wang et al., 2021). Its common pathogen has also reduced the production of apple and guava, causing considerable economic losses (Harteveld et al., 2014; Solarte et al., 2018). Tea gray blight is instigated by *Pestalotiopsis*-like species; the infection appears initially as small brown marks on the leaves and develops into large round brown necrotic lesions, severely affecting the quality of tea production.

In this study, *Pseudopestalotiopsis camelliae-sinensis* (*Pcs*) was isolated from tea gray blight, and its cytological characteristics were investigated before pathogenicity tests. Transcriptomic analysis was performed on *Pcs*-infected tea plants, the *C. sinensis* UDP-glucosyltransferase (UGT) *Cs*UGT87E7 was identified, and its expression was shown to be strongly induced by infection. In vitro assays showed that *Cs*UGT87E7 catalyzes the glucosylation of SA, and its main product was identified as SGE. Downregulation of *CsUGT87E7* expression reduced the accumulation of SGE and increased the susceptibility of tea plants to pathogen infection. After infection, *CsUGT87E7*-silenced tea leaves accumulated significantly less SA and showed lower expression of PR genes. These results suggest that *Cs*UGT87E7 plays a key role in plant disease resistance through a mechanism distinct from that previously described in Arabidopsis. This study provides insight into SA metabolism and highlights the role of *Cs*UGT87E7 in the formation of SGE and the modulation of plant defense.

## Results

### Pathogen isolation and pathogenicity analysis

**Figure 1:**
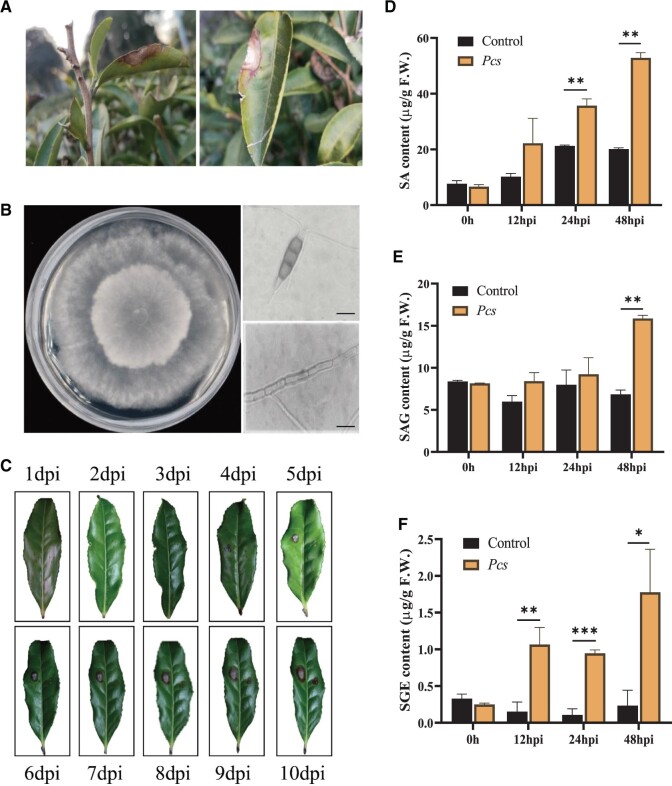
Isolation of pathogenic fungus from diseased leaves and the levels of SA and two glucosylated SA forms in tea plants after fungal infection. A, Gray blight disease of tea plants in the field. B, The plant pathogenic fungus *Pcs* isolated from diseased tea leaves and grown on potato dextrose agar (PDA). Photos on the right show conidia and fungal hyphae. Bar = 20 µm. C, Pathogenicity test of fungi on tea plant leaves. D, The relative content of SA after *Pcs* infection. E and F, The relative contents of SAG and SGESGE after *Pcs* infection. Control, inoculated with pure water. Data are presented as mean ± sd of at least three replicates. Significant differences between the treatment and the control group were calculated by one-way ANOVA in SPSS 21.0. *P <* *0.05, **P <* *0.01, and ***P <* *0.001.

Tea plants with typical gray blight disease symptoms (concentric gray rings with scattered black granules) under field conditions were selected for pathogen isolation and identification (Figure  1, A). The pathogen was purified from the diseased tea leaves and identified to the species level based on its morphological (Figure  1, B) and molecular characteristics. Genomic DNA was extracted from the purified pathogen and amplified, and the internal transcribed spacer (ITS) gene region was sequenced using primers ITS1 and ITS4 (White et al., 1990). The ITS sequence of the pathogen showed 99% similarity with *Pcs* sequences. We therefore confirmed that the fungus was *Pcs* based on its morphological and molecular characteristics.

The pathogenicity of the *Pcs* isolate was confirmed using Koch’s postulates. The leaves of healthy tea plants developed visible gray blight symptoms after inoculation with the *Pcs* strain. Five to ten days after inoculation, disease symptoms were noted on the epidermal surface, including extensive cell necrosis and lesion development (Figure  1, C). These symptoms were consistent with the symptoms observed in the above-mentioned gray blight disease under field conditions (Figure  1, A). Therefore, Koch’s postulates confirmed that the pathogen re-isolated from the diseased tea leaves was the *Pcs* pathogenic fungus.

### Fungal infection triggers the formation of SGE

It has been proposed that SA acts as an endogenous signal responsible for inducing SAR in plants (Gaffney et al., 1993). We therefore measured SA content by liquid chromatography–mass spectrometry (LC–MS) in tea plants after *Pcs* pathogen infection. The SA content increased continuously in infected plants (about 1.7- to 2.6-fold) compared with control plants that were inoculated with water (Figure  1, D).

Endogenous or exogenous SA is metabolized to a glucose conjugate in plants (Lee and Raskin, 1998). To investigate the formation and metabolism of SA after *Pcs* infection, we measured levels of the SA glucose conjugates SAG and SGE. The SAG content of infected tea plants remained at relatively stable levels within 24 hpi and its content significantly increased at 48 hpi compared with that in the control plants (Figure  1, E). SGE was almost undetectable in the healthy tea plants, but it was significantly induced at all selected time points by pathogenic fungus infection (Figure  1, F). Taken together, our results showed that SA is mainly metabolized and stored as SAG in healthy tea plants, whereas SGE formation was influenced earlier than SAG in response to fungal infection. These results suggest that SGE might play some role(s) in the defense against pathogen invasion and motivated us to investigate its formation and function in response to *Pcs* infection in tea plants.

### *Cs*UGT87E7 catalyzes the formation of SGE in vitro

**Figure 2:**
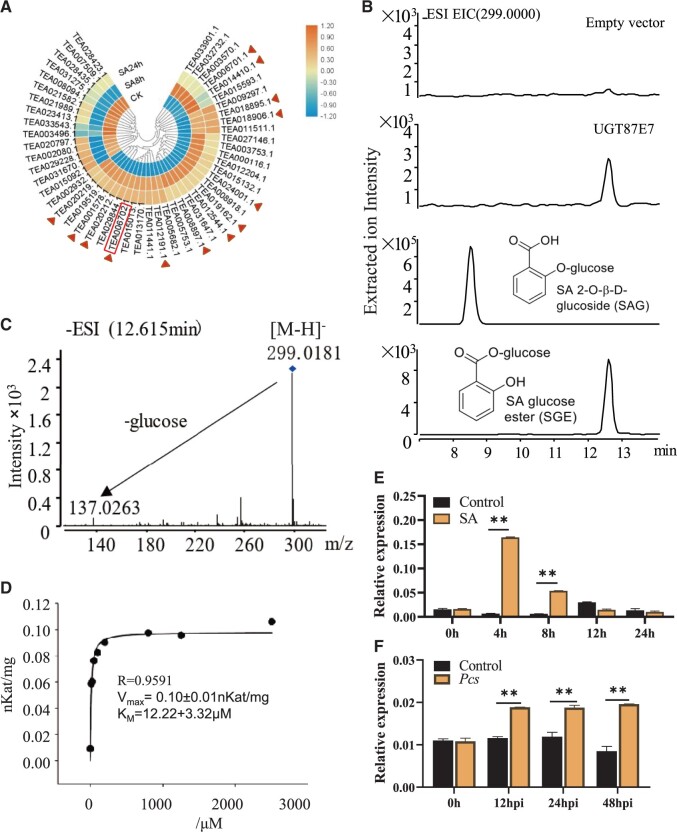
CsUGT87E7 catalyzed the glycosylation of SA in vitro and induced by SA treatment and fungal infection in tea plants. A, SA-responsive UGT genes identified in the tea plant transcriptome after SA treatment. Cloned genes are marked by a triangle and *Cs*UGT87E7 characterized in this study is boxed. B, The enzymatic reaction product was detected by LC–MS in negative ion mode, using an authentic standard and the empty vector as controls. C, Mass spectrum of the product formed by *Cs*UGT87E7. D, The kinetics of *Cs*UGT87E7 was measured under optimized conditions of reaction time. E and F, RT-qPCR analysis of *Cs*UGT87E7 relative expression level induced by SA treatment (E) and by fungal infection (F). The *GAPDH* was used as the internal reference gene. Data are presented as mean ± sd of at least three biological replicates. Significant differences between the treatment and the control group were calculated by one-way ANOVA in SPSS 21.0. *P <* *0.05, **P <* *0.01.

The transcription of glycosyltransferase genes is typically induced by their substrates (Huang et al., 2018; Jing et al., 2019; Zhao et al., 2020)*.* To search for UGTs associated with SGE formation, we performed transcriptome sequencing from 0 to 48 h after the application of exogenous SA. Tea plants sprayed with water served as controls. Transcript levels of putative UGTs were analyzed and 13 UGTs whose expression was substantially induced by SA application were selected as candidates (Figure  2, A). The full-length sequences of these candidate UGTs were obtained from young leaves of *C. sinensis* cv. Shuchazao. All UGTs were successfully expressed in *Escherichia* *coli* BL21, affinity purified, and used for subsequent enzymatic activity analysis (Supplemental Table S1). The products were analyzed by LC–MS.

The UGT encoded by *Tea006702* catalyzed the formation of SA glycoside, as identified by LC–MS. In negative ionization mode, the dominant ion peaks at *m/z* 137.02 (M-H^+^-Glc) and 299.01 (M-H^+^) were compared with authentic SGE and SAG standards (Figure  2, B and C). The results showed that the protein encoded by *Tea006702* catalyzed the formation of SGE, and it was assigned the name *Cs*UGT87E7 based on the conventions of the UGT Nomenclature Committee (Mackenzie et al., 1997).

### Substrate specificity and kinetic constants of *Cs*UGT87E7

The enzymatic activity of *Cs*UGT87E7 was further analyzed by a UDP-GLO glycosyltransferase assay using a broad range of chemical classes, including phytohormones (ABA, IBA, JA, and IAA), flavonols (kaempferol, quercetin, and naringenin), and carboxylic acids (sorbic acid and benzoic acid). UDP-glucose served as the donor substrate. *Cs*UGT87E7 did not catalyze the glucosylation of any of these substrates. Furthermore, sugar donor analysis clearly revealed that *Cs*UGT87E7 preferred UDP-Glc (100%) to UDP-Gal, UDP-GA, and UDP-Rha as sugar donor (Supplemental Figure S2).

To determine its kinetic constants, *Cs*UGT87E assay conditions were established using SA and UDP-glucose as the acceptor and donor substrate, respectively. Kinetic properties were determined for SA in the linear range of the enzymatic reaction (2 µg of protein, 30 min reaction time) at optimized conditions (Supplemental Figures S3–S5), and the apparent K_M_ value of *Cs*UGT87E7 for SA was determined to be 12.22 ± 3.22 µM (Figure  2, D). The low K_m_ value for SA strongly suggests that SA is the in vivo substrate of *Cs*UGT87E7. These results indicate that *Cs*UGT87E7 is one of the enzymes involved in the formation of SGE.

### Expression of *CsUGT87E7* is induced by exogenous SA and *Pcs*

**Figure 3:**
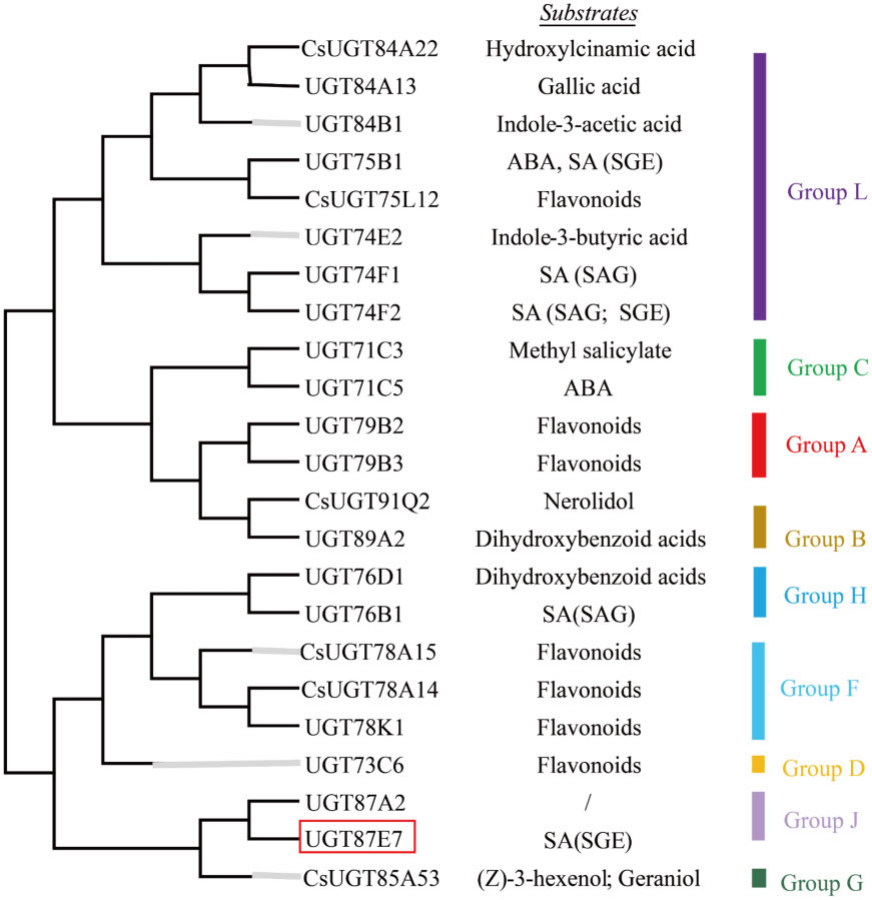
Phylogenetic analysis of *Cs*UGT87E7 and other identified functional UGTs. The phylogenetic tree was constructed by the maximum-likelihood method in MEGA 7.0 using 1,000 bootstrap replicates. Proteins selected in this study are boxed. ABA, abscisic acid.

To determine whether *CsUGT87E7* expression was induced by pathogen attack, tea leaves were exposed to a time course of *Pcs* and SA treatments, and reverse transcription quantitative PCR (RT–qPCR) was used to analyze the relative *CsUGT87E7* expression levels. As shown in Figure  2, E and F, *CsUGT87E7* was significantly induced by SA application and *Pcs* infection. Phylogenetic analysis showed that *Cs*UGT87E7 in tea plants belongs to group J (Figure  3). These results indicate that *Cs*UGT87E7 is a pathogen-responsive gene and probably plays a role in tea plant defense.

### *Cs*UGT87E7 catalyzes the formation of SGE in vivo

**Figure 4:**
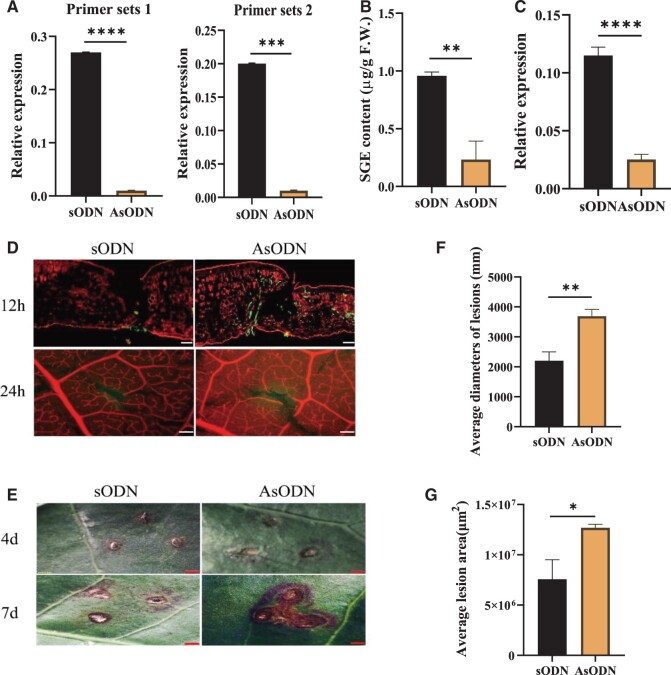
CsUGT87E7 expression and SA glucoside accumulation in tea plants after *CsUGT87E7* suppression and the disease phenotypes of fungal infection in *CsUGT87E7-*silenced and control tea plants. A, The RT-qPCR results of *CsUGT87E7* suppression in tea plants at 12 h obtained using primer sets 1 and 2 (refer to Supplemental Table S2). B, Quantitative analysis of SA glucoside ester after *CsUGT87E7* suppression. C, The relative expression level of *Cs*UGT8*7*E7 after *CsUGT87E7* suppression after 4 d. D. WGA staining results at 12 and 24 h after *Pcs* infection. Infected fungi are indicated by green staining. Bar = 50 µm at 12 h; Bar = 500 µm at 24 h. E, Disease symptoms after fungal infection were observed under a stereomicroscope. Bar = 1 mm. F, The average lesion diameter was calculated at 4 dpi. G, The average lesion area was calculated at 7 dpi. Data are presented as mean ± sd of three biological replicates (each with three technical replicates). Significant differences between the treatment and the control group were calculated by one‐way ANOVA in SPSS 21.0. **P <* *0.01, *P <* *0.05.

To further determine whether *Cs*UGT87E7 was involved in SGE formation in the tea plant, its expression was transiently suppressed in tea leaves by the gene-specific antisense oligodeoxynucleotide suppression (AsODN) strategy, as described in Zhao et al. (2019). *CsUGT87E7* expression was significantly lower in tea leaves treated with AsODN_*Cs*UGT87E7 for 12 h than in control leaves treated with sense oligonucleotides (sODN), as measured with two independent primers (Figure  4, A).

Next, the SGE content of tea leaves treated with AsODN_*Cs*UGT87E7 was measured by LC–MS. The accumulation of SGE was markedly lower in *CsUGT87E7*-silenced tea leaves (*P *<* *0.01) compared with control leaves (Figure  4, B), suggesting that *Cs*UGT87E7 is involved in the formation of SGE in vivo.

### *Cs*UGT87E7 positively modulates disease resistance of tea plants

Given that *Cs*UGT87E7 could glucosylate SA to form SGE in vivo, the next question was whether *Cs*UGT87E7 was involved in plant defense. To investigate this possibility, we first suppressed the *CsUGT87E7* expression of tea seedlings by injecting 1 mL of 10 µM AsODN-*Cs*UGT87E7 solution; control seedlings were injected with sODN. RT-qPCR analysis showed that the relative expression of *CsUGT87E7* could be silenced for at least 4 d (Figure  4, C). The seedlings were then inoculated with *Pcs*, and their phenotypes were observed for 1 week.

Although there were no differences on the leaf surfaces of tea plants with and without *Pcs* infection (Supplemental Figure S6), fungal invasion was more severe in the *CsUGT87E7*-silenced tea plants than in the controls in the first day, as observed by wheat germ agglutinin (WGA) staining (Figure  4, D). Four days after inoculation, disease symptoms became more severe in the *CsUGT87E7*-silenced plants than in the controls, and symptom severity continued to increase at 7 d after inoculation (Figure  4, E). The average lesion diameters of tea leaves at 4 d post-inoculation (dpi) (Figure  4, F) and the lesion areas of tea leaves at 7 dpi (Figure  4, G) were calculated by examination under a stereo microscope. The average lesion area was greater in *CsUGT87E7*-silenced leaves than in control leaves, indicating that *Cs*UGT87E7 positively modulates defense against *Pcs* in tea plants.

### *Cs*UGT87E7 affects SA and reactive oxygen species accumulation in infected tea leaves

**Figure 5:**
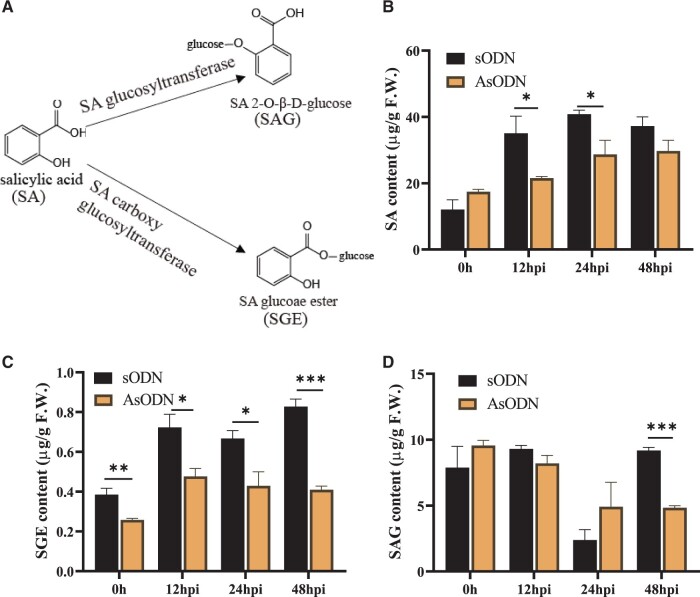
Contents of SA and two glucosylated SA forms after fungal infection in control (sODN) and *CsUGT87E7*-silenced (AsODN) tea plants. A, Chemical structures of SA, SAG, and SGE. The relative content of SA (B), SGE (C), and SAG (D) after fungal infection in control and *CsUGT87E7*-silenced tea plants. Data are presented as the mean ± sd of at least three biological replicates each representing a single infected tea plant. Significant differences between the treatment and the control group were calculated by one‐way ANOVA using SPSS 21.0. *P <* *0.05, **P <* *0.01.

In plants, the glucose conjugate of SA can exist as either SAG or SGE (Figure  5, A). To further verify the role of *Cs*UGT87E7-mediated SGE formation in the tea immune response, we inoculated *CsUGT87E7*-silenced tea plants (AsODN) and control tea plants with a spore suspension of *Pcs* and measured SA content (Figure  5, B). As shown in Figure  5, C, the content of SGE was significantly reduced in *CsUGT87E7*-silenced plants to around 35%–50% of that in the controls during pathogen infection. The SAG content was not changed and even increased at 24 h post inoculation (hpi) by pathogen (Figure  5, D), indicating that the metabolite flux of SA was shifted to SAG formation due to the silencing of *CsUGT87E7*. The reduction of SAG formation after 48 h is probably caused by the reduced SA accumulation (Figure  5, B). Taken together, it is clearly proven that *Cs*UGT87E7 plays a key role in the formation of SGE.

**Figure 6:**
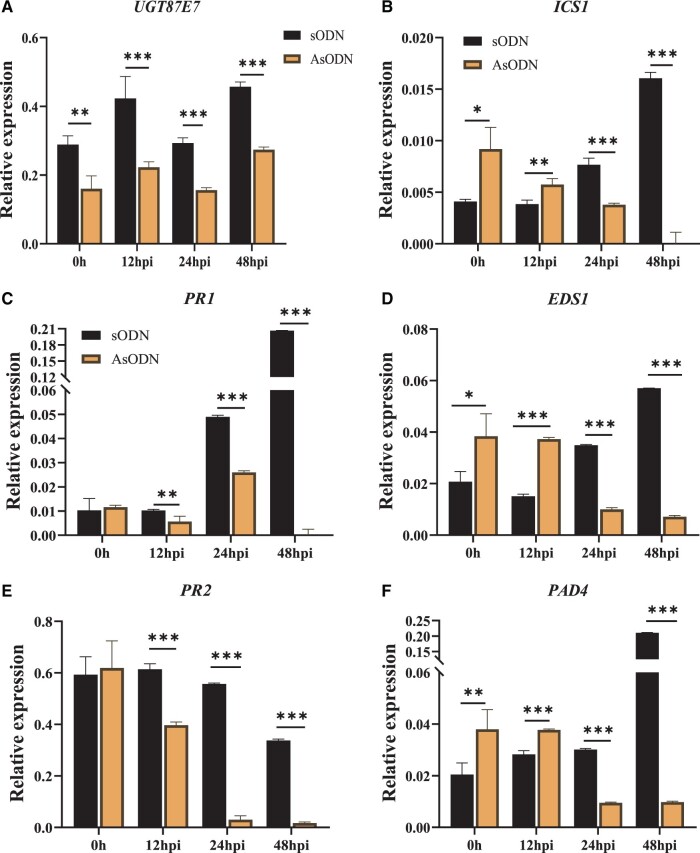
The relative expression levels of *CsUGT87E7 and* PR genes in fungus-infected leaves of control (sODN) and *CsUGT87E7*-silenced (AsODN) tea plants. A, Relative expression levels of *CsUGT87E7* in treated tea plants at different time points. B, Relative expression of SA biosynthesis genes. C, Relative expression of *PR1* and *PR2*. *GAPDH* was used as the reference gene. Data are presented as the mean ± sd of at least three biological replicates each representing a single infected tea plant. Significant differences between the treatment and the control group were calculated by one‐way ANOVA using SPSS 21.0. *P < 0.05, **P < 0.01.

The SA biosynthesis gene *ICS1* (*Isochorismate synthase 1*), the SA biosynthesis regulatory genes *EDS1* (*Enhanced disease susceptibility 1*) and *PAD4* (*Phytoalexin-deficient 4*), and the PR protein genes *PR1* and *PR2* (Maleck et al., 2000) were studied in the infected leaves in both *CsUGT87E7*-silenced and control tea plants after pathogen infection. We first measured the effect of gene suppression at each sampling time to confirm that *CsUGT87E7* was effectively silenced in the tea plants (Figure  6, A). We found that the expression levels of the upstream genes *ICSI*, *EDS1*, and *PAD4* were higher at 0 (without inoculation) and 12 hpi, whereas their expression was significantly reduced in *CsUGT87E7*-silenced plants when compared with the controls 24 h after infection (Figure  6, B), indicating that silencing of *CsUGT87E7* affects SA biosynthesis in infected leaves of tea plants during pathogen infection. It should be noted that the expression levels of downstream genes such as *PR1* and *PR2* were markedly reduced in *CsUGT87E7*-silenced plants when compared with the controls from the earliest time point after infection (Figure  6, C). Together, these results suggest that *Cs*UGT87E7-mediated SGE formation influences SA homeostasis and disease resistance during pathogen infection.

**Figure 7:**
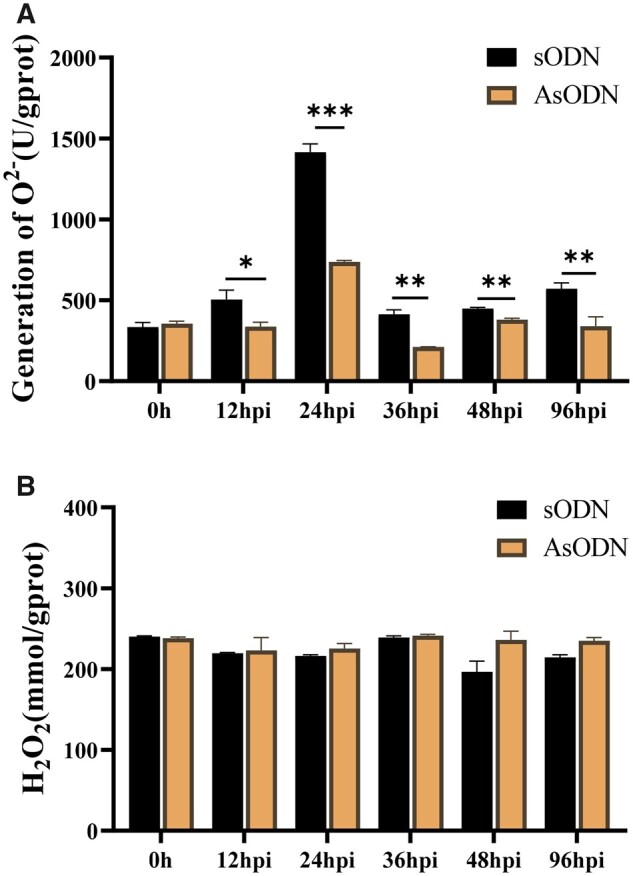
Variation of ROS after fungal infection in control (sODN) and *CsUGT87E7*-silenced (AsODN) tea plants. A, Generation of $O_{2}^{•–}$ after fungal infection in treated tea plant. B, The content of H_2_O_2_ after fungal infection in treated tea plant. Data are presented as the mean ± sd of at least three biological replicates (each with three technical replicates). Significant differences between the treatment and the control group were calculated by one‐way ANOVA using SPSS 21.0. *P < 0.05, **P < 0.01.

Reactive oxygen species (ROS) plays a very important role in plant disease resistance, it accumulates rapidly to kill pathogens (Low and Merida, 1996). SA increases ROS levels by inhibiting the activities of ROS-scavenging enzymes (Chen et al., 1993; Durner and Klessig, 1995). To investigate if *Cs*UGT87E7 affects ROS levels, we directly measured the accumulation of ROS. The generation of superoxide anion ($O_{2}^{•–}$) in *CsUGT87E7*-silenced tea plants was clearly lower than that in the control plants at all selected time points after inoculation (Figure  7, A). In contrast, the H_2_O_2_ content is similar in both *CsUGT87E7*-silenced and control plants (Figure  7, B), indicating that the expression of *CsUGT87E7* might affect the accumulation of $O_{2}^{•–}$ to activate tea plant immune response.

## Discussion

### *Cs*UGT87E7 is a SA carboxy glucosyltransferase

SA exists in plants in free acid and conjugated forms, metabolism is mainly through glucosylation, and methylation or hydroxylation (Lee et al., 1995; Dempsey et al., 2011). Glycosylation of hydroxylated SA (2,3-DHBA and 2,5-DHBA) and methylated SA (MeSA) has been studied in recent years (Zhang et al., 2017; Huang et al., 2018; Chen et al., 2019). Two enzymes (UGT74F1 and UGT74F2) were identified as SA glucosyltransferases among 90 UGTs in Arabidopsis (Lim et al., 2002). UGT74F1 forms SAG, whereas UGT74F2 forms both SAG and SGE from SA and UDP-glucose (Lim et al., 2002; Dean and Delaney, 2008). Recently, UGT76B1 was identified as a novel SAGT in Arabidopsis and shown to catalyze the conversion of SA to SAG (Noutoshi et al., 2012). Although UGT75B1 forms a glucose ester with the carboxyl group of BA, 3-HBA, 4-HBA, and 3, 4-DHBA, very little activity was observed toward SA (Lim et al., 2002). Until now, a specific SA carboxy glucosyltransferase with high efficiency in converting SA to SGE has not been identified, and there have been few studies on SGE function in plants.

Numerous studies have shown that *SAGT* expression is induced by exogenous SA application or pathogen attack (Kobayashi et al., 2020). Here, we used transcriptomic analysis to identify the putative SA glucosyltransferase *Cs*UGT87E7, whose expression was significantly induced by SA application and *Pcs* infection (Figure  2, E and F). Both in vitro and in vivo data demonstrate that *Cs*UGT87E7 is a SA carboxy glucosyltransferase (Figures  2, 4). Through phylogenetic analysis (Figure  3), we found that most of the known SA glycosyltransferases, including UGT74F1 and UGT74F2, belong to group L (Lim et al., 2002), although UGT76B1 is found far from them in group H on the phylogenetic tree (Noutoshi et al., 2012). Although phylogenies can be used to predict the functions of unknown genes, some UGTs that cluster together may have very different functions. UGT75B1 and CsUGT75L12 belong to the same group, but UGT75B1 catalyzes the glycosylation of ABA and SA, whereas *Cs*UGT75L12 acts only on flavonoids (Chen et al., 2020; Eudes et al., 2008). *Cs*UGT87E7 identified in the current study belongs to group J (Figure  3), and another member of this group, UGT87A2, has been reported to regulate flowering time and to participate in plant adaptation to abiotic stresses (Li et al., 2017; Wang et al., 2012).

### SGE formation affects SA homeostasis during pathogen infection

In recent years, SA has been the focus of intensive research because of its function as an endogenous signal and its role in defense responses to pathogens. The accumulation of SA in plants is essential for plant resistance, but most SA in cells is glucosylated and/or methylated (Rivas-San Vicente and Plasencia, 2011). Most endogenous SA, induced by pathogen infection, and exogenous SA application is metabolized into SAG in *N.* *benthamiana* (Song, 2006). Like other plant hormones and growth regulators, SAG has been shown to be stored in the vacuole (Dean and Mills, 2004; Dean et al., 2005; Zhang et al., 2017), and it can be converted into SA by a β-glucosidase to serve as a signaling molecule for plant defense response (Seo et al., 1995). In plants, SAG appears to be the predominant metabolite, whereas SGE is a relatively minor metabolite (Dean and Mills, 2004; Song, 2006). Likewise, our results showed that SAG was the predominant glucose conjugate in tea and that SGE was almost undetectable in healthy plants (Figure  1, F). This result is supported by a previous study in which SA was transformed into SAG and methyl salicylate in tea plants (Li et al., 2021).

The existence of a mechanism that releases SA from SAG suggests a possible role for SAG in plant immunity (Hennig et al., 1993). It should be noted that SGE was significantly induced by pathogenic fungal infection, although it was almost undetectable in healthy tea plants (Figure  1, F). This result suggests that SGE plays some role(s) in the response to *Pcs* infection in tea. Silencing *CsUGT87E7* in tea plants led to a decrease in SGE. Interestingly, the production of SA was also much lower in *CsUGT87E7*-silenced plants than in controls, presumably the downregulation of *CsUGT87E7* can lead to decreased expression levels of SA-biosynthesis-related genes via feedback inhibition of the structural genes (Yin et al., 2012). In our previous study, downregulation of *CsUGT78A14* led to decreased expression of flavonoid-related genes, ultimately leading to reduced flavonoid content (Zhao et al., 2019). Taken together, our results show that the formation of SGE catalyzed by *Cs*UGT87E7 affects SA homeostasis during pathogen infection, which in turn influences the SA-associated plant resistance in tea plants.

### *Cs*UGT87E7 positively regulates disease resistance in tea plants

SA-associated plant disease resistance depends largely on the interplay between free and conjugated SA forms (Chivasa and Carr, 1998; Boachon et al., 2014; Ding and Ding, 2020). Generally, plants respond to pathogen infection by rapidly increasing SA content and activating PR genes (Nawrath and Métraux, 1999; Park et al., 2004; Vlot et al., 2021). Uninfected regions of the plant are induced to generate long-term resistance (Vlot et al., 2021). In this study, we showed that *CsUGT87E7* was induced by pathogen infection (Figure  2, F). In Arabidopsis, SA glucosyltransferase1 (*At*SGT1) was rapidly induced by exogenous SA and infection with a bacterial pathogen, indicating that *AtSGT1* expression is an early disease response (Song, 2006). In Arabidopsis, UGT74F1 forms only SAG, whereas UGT74F2 forms both SAG and SGE (Lim et al., 2002). The *ugt74f1* mutant showed enhanced disease susceptibility, whereas the *ugt74f2* mutant showed enhanced resistance to the same pathogen (Boachon et al., 2014) by negatively influencing the accumulation of free SA. By contrast, the expression levels of PR genes were all reduced after *Pcs* infection in *CsUGT87E7*-silenced tea plants, leading to more severe disease symptoms (Figure  4). These results indicate that *Cs*UGT87E7 positively regulates disease resistance in infected leaves in tea plants (Figure  6) by a mechanism distinct from that previously documented in Arabidopsis (Boachon et al., 2014).

**Figure 8:**
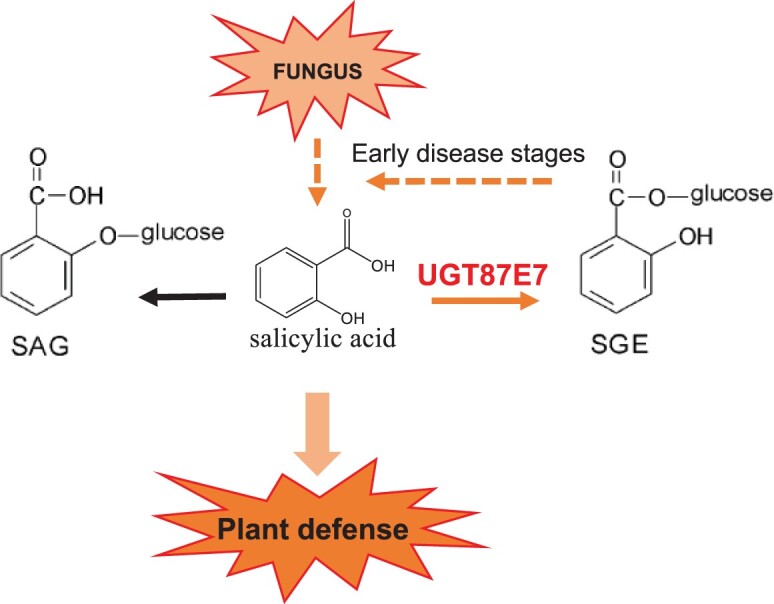
Working model of *Cs*UGT87E7 function in the regulation of SA-mediated defense in tea plant. When the tea plant is invaded by a fungus, *Cs*UGT87E7 expression is up-regulated, leading to the accumulation of SGE. The demand for free SA leads to the up-regulation of SA biosynthesis genes in the early disease stages. As a consequence, levels of pathogen-related proteins increase and plant defense are activated.

Based on these results, we propose a putative working model for the function of *Cs*UGT87E7 in pathogen infection (Figure  8). When the tea plant is invaded by a fungus, *CsUGT87E7* expression is upregulated, leading to the accumulation of SGE. The demand for free SA leads to the upregulation of SA biosynthesis genes in the early disease stages. Consequently, the elevated SA causes rapid ROS accumulation (Figure  7) by inhibiting the activities of ROS-scavenging enzymes (Chen et al., 1993). The increased ROS may act as secondary messengers to induce pathogen-related gene expression (Figure  6), and thus activates the plant disease resistance response (Figure  8). On the other hand, when the expression of *CsUGT87E7* is silenced, both SGE and SA formation decreases, dampening the SA-associated resistance response. Taken together, our findings demonstrate that *Cs*UGT87E7 is a SA carboxyl glucosyltransferase that plays a positive role in disease resistance of tea plants by modulating SA homeostasis, although the detailed mechanisms of SGE formation during plant defense require further investigation.

## Materials and methods

### Plant materials and growth conditions

Tea plant samples (*C.* *sinensis* var. *sinensis* cv. Suchazao) were collected from the horticultural research station of Anhui Agricultural University during the early spring. The samples used for metabolite and transcriptome analysis were immediately frozen in liquid nitrogen and stored at −80°C until use.

### Gene suppression of *CsUGT87E7* in tea plants

Candidate antisense oligonucleotides (AsODN) were selected using SOLIGO software (Ding and Lawrence, 2003) with *Cs*UGT87E7 as the input sequence (Supplemental Table S2). AsODNs were synthesized by the General Biosystems Company (Anhui, China). To silence *Cs*UGT87E7 in leaves that were still attached to the plant, 1 mL of 10 μM AsODN *Cs*UGT87E7 solution was injected into tea seedlings, and seedlings injected with the sODN were used as controls. After 24 h of incubation, the leaves were inoculated as described below.

### Pathogen experiments

Identified fungal isolates were cultured on PDA plates at 28°C for 4–5 d. Spores from the culture were suspended in sterile distilled water, the spores were disrupted with a sterile toothpick (Harteveld et al., 2014), and the spore suspension was adjusted to a concentration of 1× 10E7 conidia per mL using a hemocytometer. Before inoculation, the upper and lower surfaces of the leaves were disinfected with 75% alcohol, rinsed with sterile water, and air-dried. For inoculation, 50 μL of conidial suspension was inoculated onto the upper surface of the tea leaves over three needle-pierced holes. Control plants were inoculated with distilled water. Inoculated detached branches were placed in pure water in Erlenmeyer flasks and covered with plastic bags to provide continuous high humidity in the greenhouse (25°C ± 2°C) (Wang et al., 2019). To study the systemic disease resistance against *Pseudopestalotiopsis*, the lower leaves of one-year-old tea plants were primarily inoculated with fungal (*Ps.* *camelliae-sinensis*). At 24 h after primary infection, the upper leaves were collected for RNA extraction and SA analysis. Photographs of the disease resistance phenotype of both infected and systemic leaves were taken 4 d after infection of the leaves with pathogens. At least 20 leaves from different plants were assessed for each genotype during each experiment.

### RNA isolation, cDNA cloning, and sequence analysis

Total RNA was isolated from leaves of the Shuchazao cultivar using RNAiso‐mate for Plant Tissue (Takara, Dalian, China) and RNAiso Plus (Takara) according to the manufacturer’s instructions. cDNA was synthesized from total RNA by reverse transcription using HiScript III RT SuperMix for RT-qPCR (Vazyme Biotech Co. Ltd, Nanjing, China). Open-reading frame sequences were amplified using Phusion High-Fidelity DNA Polymerase (New England Biolabs, Ipswich, MA). The PCR products were purified with a Gel Extraction Kit (CWBIO, Jiangsu, China), ligated into the pGEX-4T1 vector, and subsequently transformed into Trans1T1 competent cells.

### RT-qPCR analysis

RT-qPCR was performed according to published protocols (Jing et al., 2019; Song et al., 2015) using gene-specific primers (Supplemental Table S2). The glyceraldehyde-3-phosphate dehydrogenase (GAPDH) gene was used as an internal reference gene and relative expression was calculated using the 2^–△△Ct^ method (Livak and Schmittgen, 2001). All reactions were performed on the CFX96TM System (Bio-Rad, Hercules, CA) using the temperature program 95°C for 3 min and 40 cycles of 95°C for 10 s, 62°C for 30 s.

### Heterologous protein expression and purification

The full-length sequence of *CsUGT87E7* was digested with BamH1 and Smal1, and the resulting gene fragments were subcloned into the expression vector pGEX-4T-1. The recombinant plasmids were transformed into *E. coli* BL21 (DE3) pLysS cells, which were then cultured at 37°C in Luria–Bertani (LB) liquid medium containing ampicillin (100 μg/mL) and chloramphenicol (25 μg/mL). Isopropyl-b-d-thio-galactopyranoside (IPTG, 1 mM) was added when the optical density (OD600) of the cultured cells reached 0.6–0.8. They were then incubated at 16°C and 150 rpm to permit protein expression. The fusion proteins were purified with GST·Bind resin (Jing et al., 2019) and protein concentration was determined by a photometric method (Bradford, 1976). The protein sizes were confirmed by sodium dodecyl sulfate–polyacrylamide gel electrophoresis (SDS–PAGE).

### Enzymatic activity assays

In the initial screening, each reaction mixture (25 μL in total) contained Tris–HCl buffer (50 mM, pH 7.5, 10% glycerol [v/v], and 10 mM 2-mercaptoethanol), 250 mM UDP-glucose, substrates (20 µM substrate solution), and purified protein (0.5 μg per reaction) as described in Jing et al. (2019). The reaction mixture was incubated at 30°C for 30 min and the reaction was stopped by mixing the reaction solution with the same volume of UDP-Glo assay reagent (Sheikh et al., 2017). The optimum reaction temperature and pH were determined as described in Jing et al. (2019). The optimized conditions were used for subsequent determination of kinetic parameters and at least seven different substrate concentrations and at least three biological replicates were used.

### Quantification of SA and SA glucosides

Samples were ground in liquid nitrogen and kept at −80°C before analysis. For metabolite analysis, 50 mg of sample was extracted twice with 1 mL of 75% (v/v) methanol and chlorophenylalanine solution (3 μg/mL) was added as an internal standard. The solution was mixed by vortexing and sonicated at 4°C for 20 min. After that, the mixture was centrifuged at 12,000 rpm and 4°C for 10 min. Finally, the supernatants were collected and used for LC–MS analysis (Jing et al., 2019). Products were identified by comparison of their retention time and MS spectra with those from literature or reference material. The levels of SA, SAG, and SGE were quantified by comparing the signal in the total ion chromatogram with that of the established calibration curve. SA: *y* = 709.73*x* + 528.3, *R*^2^ = 0.9988; SAG: *y* = 149.2*x*−11036, *R*^2^ = 0.9912; SGE: *y* = 126.6*x* − 30.85, *R*^2^ = 0.9931.

### Determination of ROS content in tea leaves

The hydrogen peroxide (H_2_O_2_) and superoxide anion (O2^·−^) were determined using hydrogen peroxide (H_2_O_2_) content assay kit and superoxide anion free radical (O2^·−^) content assay kit purchased from Nanjing Jiancheng Bioengineering Institute (Nanjing, China) according to the manufacturer’s instructions.

### Statistical analysis

All experiments were carried out with at least three independent biological replicates. Each measurement was carried out in triplicate. Data represent the mean ± sd of three biological replicates. Data were statistically analyzed by one-way analysis of variance (ANOVA) performed using SPSS.

### Phylogenetic tree analysis

The phylogenetic tree was constructed by the neighbor-joining method using MAGA6 software based on the deduced amino acid sequences of UGTs. The 0.1 scale bar indicates 0.1 nucleotide substitutions per site.

## Accession numbers

Sequence data from this article can be found at GenBank under accession numbers UGT74F2 (At2g43820), PR1 (At2g14610), PR2 (At3g57260), EDS1 (At3g48090), PAD4 (At3g52430), ICS1 (At1g74710), UGT74F1 (At2g43840), UGT76B1 (At3g11340), UGT75B1 (At1g05560), CsUGT78A14 (ALO19888.1), CsUGT78A15 (ALO01989.1), CsUGT75L12 (ALO19892.1), CsUGT84A22 (ALO19890), UGT89A2 (At5g03490), UGT76D1 (At2g26480), UGT71C3 (At1g07260), UGT71C5 (OAP14418.1), UGT84A13 (AHA54051.1), UGT84B1 (At2g23260), UGT74E2 (At1g05680), UGT78K1 (ADC96620.1), UGT73C6 (OAP07438.1), UGT79B2 (At4g25750), and UGT79B3 (At4g27560).

## Supplemental data

The following materials are available in the online version of this article.

**Supplemental Figure S1.** Agarose gel and SDS–PAGE of *Cs*UGT87E7.

**Supplemental Figure S2.** Glucosyltransferase activity of *Cs*UGT87E7 toward SA with different sugar donors.

**Supplemental Figure S3.** The effect of different incubation times on the product formation of *Cs*UGT87E7 using SA and UDP-glucose as substrates.

**Supplemental Figure S4.** The temperature optimization of *Cs*UGT87E7.

**Supplemental Figure S5.** The pH optimization of *Cs*UGT87E7.

**Supplemental Figure S6.** The leaf surfaces of tea plants 1 day after fungus infection.

**Supplemental Table S1.** Screening of enzyme activity toward SA.

**Supplemental Table S2.** List of primers used in this work.

## Funding

This work was supported by the National Natural Science Foundation of China (31961133030, 31870678, and 32022076), the Science Fund for Distinguished Young Scientists of Anhui Province (1908085J12), the Collaborative Innovation Project of Anhui Province (GXXT-2021-060), and the Deutsche Forschungsgemeinschaft (SCHW634/32-1 and SCHW634/34–1).

*Conflict of interest statement*. None declared.

## Supplementary Material

kiab569_Supplementary_Data

## Notes

Y.H. and C.S. designed the research. Y.H., Me.Z., Y.W., T.J., Y.Z., J.W., M.L., Z.Z., B.W., T.G., H.J., and Y.F. performed the research. Y.H., Me.Z., Mi.Z., X.W., and M.L. contributed new analytic and computational tools. Y.H., Me.Z., Mi.Z., and C.S. analyzed the data. Y.H., X.W., W.S., and C.S. wrote the manuscript. All authors agreed to the list of authors and the identified contributions of those authors.

The author responsible for distribution of materials integral to the findings presented in this article in accordance with the policy described in the Instructions for Authors (https://academic.oup.com/plphys/pages/General-Instructions) is: Chuankui Song (sckfriend@163.com).

## References

[kiab569-B72] Attaran E , Zeier TE, Griebel T, Zeier J (2009) Methyl salicylate production and jasmonate signaling are not essential for systemic acquired resistance in Arabidopsis. Plant Cell 21: 954–971 19329558 10.1105/tpc.108.063164 PMC2671706

[kiab569-B1] Bauer S , Mekonnen DW, Hartmann M, Yildiz I, Janowski R, Lange B, Geist B, Zeier J, Schäffner AR (2021) UGT76B1, a promiscuous hub of small molecule-based immune signaling, glucosylates *N*-hydroxypipecolic acid, and balances plant immunity. Plant Cell 33**:** 714–734 33955482 10.1093/plcell/koaa044 PMC8136890

[kiab569-B2] Boachon Gamir J , Pastor V, Erb M, Dean JV, Flors V, Mauch-Mani B (2014) Role of two UDP-glycosyltransferases from the L group of Arabidopsis in resistance against pseudomonas syringae. Eur J Plant Pathol 139**:** 707–720

[kiab569-B3] Bowles D , Lim EK, Poppenberger B, Vaistij FE (2006) Glycosyltransferases of lipophilic small molecules. Annu Rev Plant Biol 57**:** 567–597 16669774 10.1146/annurev.arplant.57.032905.105429

[kiab569-B4] Bradford M (1976) A rapid and sensitive method for the quantitation of microgram quantities of protein utilizing the principles of protein-dye binding. Anal Biochem 71**:** 248–254 10.1016/0003-2697(76)90527-3 942051

[kiab569-B5] Cai J , Jozwiak A, Holoidovsky L, Meijler MM, Meir S, Rogachev I, Aharoni A (2021) Glycosylation of *N*-hydroxy-pipecolic acid equilibrates between systemic acquired resistance response and plant growth. Mol Plant 14**:** 440–455 33387676 10.1016/j.molp.2020.12.018

[kiab569-B6] Chanda B , Xia Y, Mandal MK, Yu K, Sekine KT, Gao QM, Selote D, Hu Y, Stromberg A, Navarre D, et al (2011) Glycerol-3-phosphate is a critical mobile inducer of systemic immunity in plants. Nat Genet 43**:** 421–427 21441932 10.1038/ng.798

[kiab569-B7] Chaturvedi R , Krothapalli K, Makandar R, Nandi A, Sparks AA, Roth MR, Welti R, Shah J (2008) Plastid omega3-fatty acid desaturase dependent accumulation of a systemic acquired resistance inducing activity in petiole exudates of *Arabidopsis thaliana* is independent of jasmonic acid. Plant J 54**:** 106–117 18088304 10.1111/j.1365-313X.2007.03400.x

[kiab569-B8] Chen L , Wang WS, Wang T, Meng XF, Chen TT, Huang XX, Li YJ, Hou BK (2019) Methyl salicylate glucosylation regulates plant defense signaling and systemic acquired resistance. Plant Physiol 180**:** 2167–2181 30962291 10.1104/pp.19.00091 PMC6670094

[kiab569-B9] Chen TT , Liu FF, Xiao DW, Jiang XY, Li P, Zhao SM, Hou BK, Li YJ (2020) The Arabidopsis UDP-glycosyltransferase 75B1, conjugates abscisic acid and affects plant response to abiotic stresses. Plant Mol Biol 102**:** 389–401 31894456 10.1007/s11103-019-00953-4

[kiab569-B10] Chen YC , Holmes EC, Rajniak J, Kim JG, Tang S, Fischer CR, Mudgett MB, Sattely ES (2018) *N*-hydroxy-pipecolic acid is a mobile metabolite that induces systemic disease resistance in Arabidopsis. Proc Natl Acad Sci USA 115**:** 4920–4929 10.1073/pnas.1805291115 PMC6003486 29735713

[kiab569-B11] Chen Z , Malamy J, Henning J, Conrath U, Sanchez-Casas P, Silva H, Ricigliano J, Klessig DK (1995). Induction, modification, and transduction of the salicylic acid signal in plant defense responses. Proc Natl Acad Sci USA 92**:** 4134–4137 11607539 10.1073/pnas.92.10.4134 PMC41899

[kiab569-B12] Chen Z , Silva H, Klessig DF (1993). Active oxygen species in the induction of plant systemic acquired resistance by salicylic acid. Science 262**:** 1883–1886 8266079 10.1126/science.8266079

[kiab569-B13] Chisholm ST , Coaker G, Day B, Staskawicz BJ (2006) Host–microbe interactions: shaping the evolution of the plant immune response. Cell 124**:** 803–814 16497589 10.1016/j.cell.2006.02.008

[kiab569-B75] Chivasa, Carr (1998) Cyanide restores N gene-mediated resistance to tobacco mosaic virus in transgenic tobacco expressing salicylic acid hydroxylase. Plant Cell 10: 1489–1498 9724695 10.1105/tpc.10.9.1489 PMC144082

[kiab569-B70] Choudhary DK , Prakash A, Johri BN (2007) Induced systemic resistance (ISR) in plants: mechanism of action. Indian J Microbiol 47: 289–297 23100680 10.1007/s12088-007-0054-2 PMC3450033

[kiab569-B69] Conrath U , Beckers GJ, Langenbach CJ, Jaskiewicz MR (2015) Priming for enhanced defense. Annu Rev Phytopathol 53: 97–119 26070330 10.1146/annurev-phyto-080614-120132

[kiab569-B14] Dean JV , Delaney SP (2008) Metabolism of salicylic acid in wild-type, ugt74f1 and ugt74f2 glucosyltransferase mutants of *Arabidopsis thaliana*. Physiol Plant 132**:** 417–425 18248508 10.1111/j.1399-3054.2007.01041.x

[kiab569-B15] Dean JV , Mills JD (2004) Uptake of salicylic acid 2-*O*-beta-d-glucose into soybean tonoplast vesicles by an ATP-binding cassette transporter-type mechanism. Physiol Plant 120**:** 603–612 15032822 10.1111/j.0031-9317.2004.0263.x

[kiab569-B85] Dean JV , Mohammed LA, Fitzpatrick T (2005) The formation, vacuolar localization, and tonoplast transport of salicylic acid glucose conjugates in tobacco cell suspension cultures. Planta 221: 287–296 15871031 10.1007/s00425-004-1430-3

[kiab569-B77] Dempsey DA , Vlot AC, Wildermuth MC, Klessig DF (2011) Salicylic Acid Biosynthesis and Metabolism. Arab B 9: 1–24 10.1199/tab.0156 PMC3268552 22303280

[kiab569-B16] Ding Y , Lawrence CE (2003) A statistical sampling algorithm for RNA secondary structure prediction. Nucleic Acids Res 31**:** 7280–7301 14654704 10.1093/nar/gkg938 PMC297010

[kiab569-B17] Ding P , Ding Y (2020) Stories of salicylic acid: A plant defense hormone. Trends Plant Sci 25**:** 549–565 32407695 10.1016/j.tplants.2020.01.004

[kiab569-B18] Durner J , Klessig DF (1995) Inhibition of ascorbate peroxidase by salicylic acid and 2, 6-dichloroisonicotinic acid, two inducers of plant defense responses. Proc Natl Acad Sci USA 92**:** 11312–11316 7479986 10.1073/pnas.92.24.11312 PMC40622

[kiab569-B19] Durrant WE , Dong X (2004) Systemic acquired resistance. Annu Rev Phytopathol 42**:** 185–209 15283665 10.1146/annurev.phyto.42.040803.140421

[kiab569-B22] Enyedi AJ , Yalpani N, Silverman P, Raskin I (1992) Localization, conjugation, and function of salicylic acid in tobacco during the hypersensitive reaction to tobacco mosaic virus. Proc Natl Acad Sci USA 89**:** 2480–2484 1549613 10.1073/pnas.89.6.2480 PMC48682

[kiab569-B23] Eudes A , Bozzo GG, Waller JC, Naponelli V, Lim EK, Bowles DJ, Gregory JF 3rd, Hanson AD (2008) Metabolism of the folate precursor p-aminobenzoate in plants: glucose ester formation and vacuolar storage. J Biol Chem 283**:** 15451–15459 18385129 10.1074/jbc.M709591200 PMC2397476

[kiab569-B79] Gaffney T , Friedrich L, Vernooij B, Negrotto D, Nye G, Uknes S, Ward E, Kessmann H, Ryals J (1993) Requirement of salicylic acid for the induction of systemic acquired resistance. Science 261: 754–756 17757215 10.1126/science.261.5122.754

[kiab569-B26] Harteveld DOC , Akinsanmi OA, Drenth A (2014) Pathogenic variation of *Alternaria* species associated with leaf blotch and fruit spot of apple in Australia. Eur J Plant Pathol 139**:** 789–799

[kiab569-B27] Hartmann M , Zeier T, Bernsdorff F, Reichel-Deland V, Kim D, hohmann M, Scholten N, Schuck S, Bräutigam A, Hölzel T, et al (2018) Flavin monooxygenase-generated *N*-hydroxypipecolic acid is a critical element of plant systemic immunity. Cell 173**:** 456–469 29576453 10.1016/j.cell.2018.02.049

[kiab569-B28] Hennig J , Malamy J, Grynkiewicz G, Indulski J, Klessig DF (1993) Interconversion of the salicylic acid signal and its glucoside in tobacco. Plant J 4**:** 593–600 8252063 10.1046/j.1365-313x.1993.04040593.x

[kiab569-B29] Hõrak H (2021) How to achieve immune balance and harmony: glycosyltransferase UGT76B1 inactivates *N*-hydroxy-pipecolic acid to suppress defense responses. Plant Cell 33**:** 453–454 35234939 10.1093/plcell/koaa053 PMC8136871

[kiab569-B30] Huang XX , Zhu GQ, Liu Q, Chen L, Li YJ, Hou BK (2018) Modulation of plant salicylic acid-associated immune responses via glycosylation of dihydroxybenzoic acids. Plant Physiol 176**:** 3103–3119 29483147 10.1104/pp.17.01530 PMC5884596

[kiab569-B31] Jiang X , Shi Y, Dai X, Zhuang J, Fu Z, Zhao X, Liu Y, Gao L, Xia T (2018) Four flavonoid glycosyltransferases present in tea overexpressed in model plants *Arabidopsis thaliana* and *Nicotiana tabacum* for functional identification. J Chromatogr B Anal Tech Biomed Life Sci 1100–1101**:** 148–157 10.1016/j.jchromb.2018.09.033 30317153

[kiab569-B32] Jing T , Zhang N, Gao T, Zhao M, Jin J, Chen Y, Xu M, Wan X, Schwab W, Song C (2019) Glucosylation of (Z)-3-hexenol informs intraspecies interactions in plants: A case study in *Camellia sinensis*. Plant Cell Environ 42**:** 1352–1367 30421786 10.1111/pce.13479

[kiab569-B33] Jones JD , Dangl JL (2006) The plant immune system. Nature 444**:** 323–329 17108957 10.1038/nature05286

[kiab569-B34] Jung HW , Tschaplinski TJ, Wang L, Glazebrook J, Greenberg JT (2009) Priming in systemic plant immunity. Science 324**:** 89–91 19342588 10.1126/science.1170025

[kiab569-B35] Kobayashi Y , Fukuzawa N, Hyodo A, Kim H, Mashiyama S, Ogihara T, Yoshioka H, Matsuura H, Masuta C, Matsumura T, et al (2020) Role of salicylic acid glucosyltransferase in balancing growth and defence for optimum plant fitness. Mol Plant Pathol 21**:** 429–442 31965700 10.1111/mpp.12906 PMC7036366

[kiab569-B36] Lee HI , Leon J, Raskin I (1995) Biosynthesis and metabolism of salicylic acid. Proc Natl Acad Sci USA 92**:** 4076–4079 11607533 10.1073/pnas.92.10.4076 PMC41889

[kiab569-B37] Lee HI , Raskin I (1998) Glucosylation of salicylic acid in *Nicotiana tabacum* Cv. Xanthi-nc. Phytopathology 88**:** 692–697 18944942 10.1094/PHYTO.1998.88.7.692

[kiab569-B39] Li P , Li YJ, Wang B, Yu HM, Li Q, Hou BK (2017) The Arabidopsis UGT87A2, a stress-inducible family 1 glycosyltransferase, is involved in the plant adaptation to abiotic stresses. Physiol Plant 159**:** 416–432 27747895 10.1111/ppl.12520

[kiab569-B76] Li X , Lin H, Zhang W, Zou Y, Zhang J, Tang X, Zhou JM (2005) Flagellin induces innate immunity in nonhost interactions that is suppressed by Pseudomonas syringae effectors. Proc Natl Acad Sci U S A 102: 12990–12995 16123135 10.1073/pnas.0502425102 PMC1200263

[kiab569-B86] Li J , Xiao Y, Fan Q, Liao Y, Wang X, Fu X, Gu D, Chen Y, Zhou B, Tang J, et al (2021) Transformation of salicylic acid and its distribution in tea plants (*Camellia sinensis*) at the tissue and subcellular levels. Plants 10: 282 33540509 10.3390/plants10020282 PMC7912924

[kiab569-B40] Lim EK , Doucet CJ, Li Y, Elias L, Worrall D, Spencer SP, Ross J, Bowles DJ (2002) The activity of Arabidopsis glycosyltransferases toward salicylic acid, 4-hydroxybenzoic acid, and other benzoates. J Biol Chem 277**:** 586–592 11641410 10.1074/jbc.M109287200

[kiab569-B41] Lim GH , Liu H, Yu K, Liu R, Shine MB, Fernandez J, Burch-Smith T, Mobley JK, McLetchie N, Kachroo A, et al (2020) The plant cuticle regulates apoplastic transport of salicylic acid during systemic acquired resistance. Sci Adv 6**:** eaaz0478 32494705 10.1126/sciadv.aaz0478 PMC7202870

[kiab569-B42] Livak KJ , Schmittgen TD (2001) Analysis of relative gene expression data using real-time quantitative PCR and the 2(-delta delta C (T)) method. Methods 25**:** 402–408 11846609 10.1006/meth.2001.1262

[kiab569-B43] Low PS , Merida JR (1996) The oxidative burst in plant defense: Function and signal transduction. Physiol Plantarum 96**:** 533–542

[kiab569-B81] Mackenzie PI , Owens IS, Burchell B, Bock KW, Bairoch A, Bélanger A, Fournel-Gigleux S, Green M, Hum DW, Iyanagi T, et al (1997) The UDP glycosyltransferase gene superfamily: recommended nomenclature update based on evolutionary divergence. Pharmacogenetics 7: 255–269 9295054 10.1097/00008571-199708000-00001

[kiab569-B83] Maleck K , Levine A, Eulgem T, Morgan A, Schmid J, Lawton KA, Dangl JL, Dietrich RA (2000) The transcriptome of Arabidopsis thaliana during systemic acquired resistance. Nat Genet 26: 403–410 11101835 10.1038/82521

[kiab569-B44] Mohnike L , Rekhter D, Huang W, Feussner K, Tian H, Herrfurth C, Zhang Y, Feussner I (2021) The glycosyltransferase UGT76B1 modulates *N*-hydroxy-pipecolic acid homeostasis and plant immunity. Plant Cell 33**:** 735–749 33955489 10.1093/plcell/koaa045 PMC8136917

[kiab569-B45] Návarová H , Bernsdorff F, Döring AC, Zeier J (2012) Pipecolic acid, an endogenous mediator of defense amplification and priming, is a critical regulator of inducible plant immunity. Plant Cell 24**:** 5123–5141 23221596 10.1105/tpc.112.103564 PMC3556979

[kiab569-B46] Nawrath C , Métraux J-P (1999) Salicylic acid induction-deficient mutants of Arabidopsis express PR-2 and PR-5 and accumulate high levels of camalexin after pathogen inoculation. Plant Cell 11**:** 1393–1404 10449575 10.1105/tpc.11.8.1393 PMC144293

[kiab569-B47] Noutoshi Y , Okazaki M, Kida T, Nishina Y, Morishita Y, Ogawa T, Suzuki H, Shibata D, Jikumaru Y, Hanada A (2012) Novel plant immune-priming compounds identified via high-throughput chemical screening target salicylic acid glucosyltransferases in Arabidopsis. Plant Cell 24**:** 3795–3804 22960909 10.1105/tpc.112.098343 PMC3480303

[kiab569-B48] Park CH , Kim S, Park JY, Ahn IP, Lee YH (2004) Molecular characterization of a pathogenesis-related protein 8 gene encoding a class III chitinase in rice. Mol Cells 17**:** 144–150 15055541

[kiab569-B49] Park SW , Kaimoyo E, Kumar D, Mosher S, Klessig DF (2007) Methyl salicylate is a critical mobile signal for plant systemic acquired resistance. Science 318**:** 113–116 17916738 10.1126/science.1147113

[kiab569-B84] Rivas-San Vicente M , Plasencia J (2011) Salicylic acid beyond defence: Its role in plant growth and development. J Exp Bot 62: 3321–3338 21357767 10.1093/jxb/err031

[kiab569-B51] Ross AF (1961) Systemic acquired resistance induced by localized virus infections in plants. Virology 14**:** 340–358 13743578 10.1016/0042-6822(61)90319-1

[kiab569-B53] Seo S , Ishizuka K, Ohashi Y (1995) Induction of salicylic acid β-glucosidase in tobacco leaves by exogenous salicylic acid. Plant Cell Physiol 36**:** 447–453

[kiab569-B54] Shah J , Chaturvedi R, Chowdhury Z, Venables B, Petros RA (2014) Signaling by small metabolites in systemic acquired resistance. Plant J 79**:** 645–658 24506415 10.1111/tpj.12464

[kiab569-B55] Sheikh MO , Halmo SM, Patel S, Middleton D, Takeuchi H, Schafer CM, West CM, Haltiwanger RS, Avci FY, Moremen KW, et al (2017) Rapid screening of sugar-nucleotide donor specificities of putative glycosyltransferases. Glycobiology 27**:** 206–212 28177478 10.1093/glycob/cww114 PMC5789813

[kiab569-B73] Solarte F , Muñoz CG, Maharachchikumbura SSN, Álvarez E (2018) Diversity of *Neopestalotiopsis* and *Pestalotiopsis spp*., Causal Agents of Guava Scab in Colombia. Plant Dis 102: 49–59 30673452 10.1094/PDIS-01-17-0068-RE

[kiab569-B57] Song C , Gu L, Liu J, Zhao S, Hong X, Schulenburg K, Schwab W (2015) Functional characterization and substrate promiscuity of UGT71 glycosyltransferases from strawberry (*Fragaria x ananassa*). Plant Cell Physiol 56**:** 2478–2493 26454881 10.1093/pcp/pcv151

[kiab569-B58] Song JT (2006) Induction of a salicylic acid glucosyltransferase, AtSGT1, is an early disease response in *Arabidopsis thaliana*. Mol Cells 22**:** 233–238 17085977

[kiab569-B71] Thompson AMG , Iancu CV, Neet KE, Dean JV, Choe JY (2017) Differences in salicylic acid glucose conjugations by UGT74F1 and UGT74F2 from *Arabidopsis thaliana*. Sci Rep 7: 46629 28425481 10.1038/srep46629 PMC5397973

[kiab569-B61] Vlot AC , Dempsey DA, Klessig DF (2009) Salicylic acid, a multifaceted hormone to combat disease. Annu Rev Phytopathol 47: 177–206 19400653 10.1146/annurev.phyto.050908.135202

[kiab569-B62] Vlot AC , Sales JH, Lenk M, Bauer K, Brambilla A, Sommer A, Chen Y, Wenig M, Nayem S (2021) Systemic propagation of immunity in plants. New Phytol 229**:** 1234–1250 32978988 10.1111/nph.16953

[kiab569-B63] Wang B , Jin SH, Hu HQ, Sun YG, Wang YW, Han P, Hou BK (2012) UGT87A2, an Arabidopsis glycosyltransferase, regulates flowering time via flowering locus C. New Phytol 194**:** 666–675 22404750 10.1111/j.1469-8137.2012.04107.x

[kiab569-B78] Wang S , Liu L, Mi X, Zhao S, An Y, Xia X, Guo R, Wei C (2021) Multi-omics analysis to visualize the dynamic roles of defense genes in the response of tea plants to gray blight. Plant J 106: 862–875 33595875 10.1111/tpj.15203

[kiab569-B64] Wang S , Mi X, Wu Z, Zhang L, Wei C (2019) Characterization and pathogenicity of pestalotiopsis-like species associated with gray blight disease on *Camellia sinensis* in Anhui Province, China. Plant Dis 103**:** 2786–2797 31535958 10.1094/PDIS-02-19-0412-RE

[kiab569-B65] White TJ , Bruns T, Lee S, Taylor J (1990) Amplification and direct sequencing of fungal ribosomal RNA genes for phylogenetics. *In* MA Innis, DH Gelfand, JJ Sninsky, TJ White, eds, PCR Protocols, Academic Press, San Diego, pp 315–322

[kiab569-B74] Yin R , Messner B, Faus-Kessler T, Hoffmann T, Schwab W, Hajirezaei MR, von Saint Paul V, Heller W, Schäffner AR (2012) Feedback inhibition of the general phenylpropanoid and flavonol biosynthetic pathways upon a compromised flavonol-3-O-glycosylation. J Exp Bot 63: 2465–2478 22249996 10.1093/jxb/err416 PMC3346215

[kiab569-B66] Zeier J (2021) Metabolic regulation of systemic acquired resistance. Curr Opin Plant Biol 62**:** 102050 34058598 10.1016/j.pbi.2021.102050

[kiab569-B67] Zhang Y , Zhao L, Zhao J, Li Y, Wang J, Guo R, Gan S, Liu CJ, Zhang K (2017) S5H/DMR6 encodes a salicylic acid 5-hydroxylase that fine-tunes salicylic acid homeostasis. Plant Physiol 175**:** 1082–1093 28899963 10.1104/pp.17.00695 PMC5664462

[kiab569-B82] Zhao M , Gao Jin J Zhang T Jing N Wang T Ban J Schwab Q Song C (2019) Glucosyltransferase CsUGT78A14 Regulates Flavonols Accumulation and Reactive Oxygen Species Scavenging in Response to Cold Stress in *Camellia sinensis*. Front Plant Sci 10: 1–14 31929783 10.3389/fpls.2019.01675 PMC6941654

[kiab569-B68] Zhao M , Zhang N, Gao T, Jin J, Jing T, Wang J, Wu Y, Wan X, Schwab W, Song C (2020) Sesquiterpene glucosylation mediated by glucosyltransferase UGT91Q2 is involved in the modulation of cold stress tolerance in tea plants. New Phytol 226**:** 362–372 31828806 10.1111/nph.16364

