# Correction to: Salicylic acid carboxyl glucosyltransferase UGT87E7 regulates disease resistance in *Camellia sinensis*

**DOI:** 10.1093/plphys/kiac090

**Published:** 2022-03-12

**Authors:** Yunqing Hu, Mengting Zhang, Mengqian Lu, Yi Wu, Tingting Jing, Mingyue Zhao, Yifan Zhao, Yingying Feng, Jingming Wang, Ting Gao, Zixiang Zhou, Bin Wu, Hao Jiang, Xiaochun Wan, Wilfried Schwab, Chuankui Song

*Plant Physiology*, https://doi.org/10.1093/plphys/kiab569

In the originally published version of this manuscript the unit of the ROS in Fig. 7B was wrong.

The unit of Y-axis in the original Figure 7B was shown as (mol/gprot), which was incorrect. The concentrations shown in the figure were actually in µmol/gprot. The authors have revised the unit to mmol/gprot so that fewer zeros are needed in the numbers, making it easier to read. The unit of Y-axis in the original Figure 7A was also revised from U/g to U/gprot to make it consistent with that in Figure 7B.

