# Supplementary material for: Salicylic acid carboxyl glucosyltransferase UGT87E7 regulates disease resistance in Camellia sinensis
Source: Plant Physiol. 2021 Dec 6;188(3):1507–20. doi: 10.1093/plphys/kiab569 (PMC8896648; doi:10.1093/plphys/kiab569)
Supplement: kiab569_Supplementary_Data [file kiab569_supplementary_data.pdf]

# Supplemental data

**Salicylic acid carboxyl glucosyltransferase UGT87E7 regulates disease resistance in *Camellia sinensis***

Yunqing Hu[1], Mengting Zhang[1], Mengqian Lu[1], Yi Wu[1], Tingting Jing[1], Mingyue Zhao[1], Yifan Zhao[1], Yingying Feng[1], Jingming Wang[1], Ting Gao[1], Zixiang Zhou[1], Bin Wu[1], Hao Jiang, Xiaochun Wan[1], Wilfried Schwab[1, 2,†], Chuankui Song[1*,†]

[1]State Key Laboratory of Tea Plant Biology and Utilization, International Joint Laboratory on Tea Chemistry and Health Effects, Anhui Agricultural University, 230036, Hefei, Anhui, P. R. China

[2]Biotechnology of Natural Products, Technische Universität München, Liesel-Beckmann-Str. 1, 85354 Freising, Germany

*Author for communication: sckfriend@163.com
† Senior author
These authors contributed equally (Y. H. and Me.Z.).

**Supplemental Table S1** Screening of enzyme activity toward salicylic acid

| Gene sequence | Enzyme reaction with SA as substrate |
| --- | --- |
| *TEA018906.1* | - |
| *TEA020219.1* | - |
| *TEA000116.1* | - |
| *TEA006702.1* | + |
| *TEA018895.1* | - |
| *TEA024001.1* | - |
| *TEA008897.1* | - |
| *TEA014410.1* | - |
| *TEA020212.1* | - |
| *TEA019162.1* | - |
| *TEA012191.1* | - |
| *TEA012544.1* | - |
| *TEA001578.1* | - |
| *TEA029844.1* | - |
| *TEA006701.1* | - |

**Supplemental Table S2** List of primers used in this work

| Name | Sequence (5′- 3′) |
|---|---|
| For RT-qPCR analysis | |
| **Primer 1** | |
| qUGT87E7-1-F | GGGCACATCAACCCCATGAT |
| qUGT87E7-1-R | GGGCAAGACGTTTGGGATTG |
| **Primer 2** | |
| qUGT87E7-2-F | CACGTAGTGGCCATGCCTTA |
| qUGT87E7-2-R | AATCGGATGTTGTCCGGCTT |
| qPR1-F | GACAGTGTCGGATGCAGTGA |
| qPR1-R | TTCTGGCACTGAACCCTAGC |
| qPR2-F | CTCTGTTCACAGCTCCCTCG |
| qPR2-R | GTTCCACCAACTGTAGGCCA |
| qICS1-F | GAACGGCTAGCCAGTGAAGT |
| qICS1-R | AGTTGCTTCCACAGAGAGCC |
| qEDS1-F | CGACATCGTCCCTCGCATAA |
| qEDS1-R | CGGATTTGTGGACCCCATCA |
| qPAD4-F | ATGTTTCTGGGCGAGAGTGG |
| qPAD4-R | CTCCCCATTCCTTCACCCAC |
| GAPDH-F | TTGGCATCGTTGAGGGTCT |
| GAPDH-R | CAGTGGGAACACGGAAAGC |
| For gene suppression | |
| AsODN-UGT87E7-1 | TTGGTGACCCTTCATGTGGA |
| AsODN-UGT87E7-2 | GAGTTGGTGACCCTTCATGT |
| AsODN-UGT87E7-3 | ATACTCGCCGGCGTCTCTAG |
| AsODN-UGT87E7-4 | TACGATGATACTCGCCGGCG |
| For gene cloning | |
| UGT87E7-F | GGTTCCGCGTGGATCCATGGCTCCGGAAGGCG |
| UGT87E7-R | CGCTCGAGTCGACCCGGGTCATTTGGAAATATCATTGACAAAAGTATCGAG |

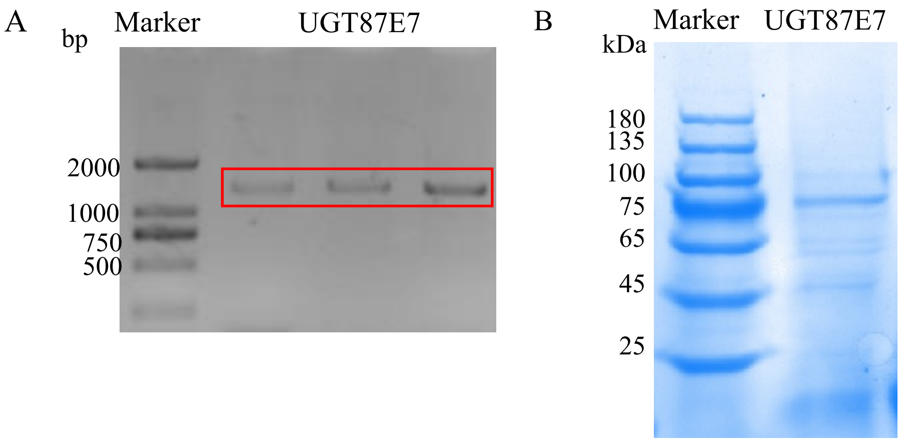


**Supplemental Figure S1.** Agarose gel and SDS-PAGE of *Cs*UGT87E7. A, The target gene length is approximately 1377 bp. B, The target protein molecular weight is approximately 75 kDa.

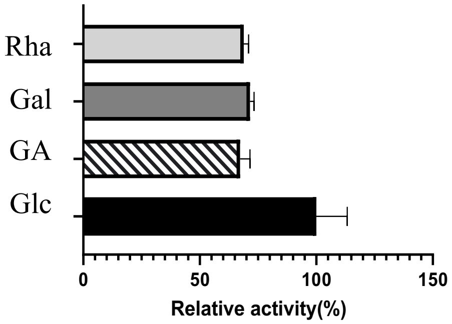


**Supplemental Figure S2** Glucosyltransferase activity of *Cs*UGT87E7 towards salicylic acid (SA) with different sugar donors. Data are presented as mean ± SD of at least three biological replicates. Rha, UDP-rhamnose; GA, UDP-galacturonic acid; Gal, UDP-galactose, Glc, UDP-glucose

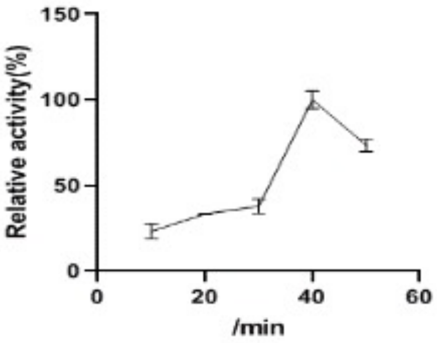


**Supplemental Figure S3** The effect of different incubation times on the product formation of *Cs*UGT87E7 using salicylic acid (SA) and UDP-glucose as substrates. Data are presented as mean ± SD of at least three biological replicates.

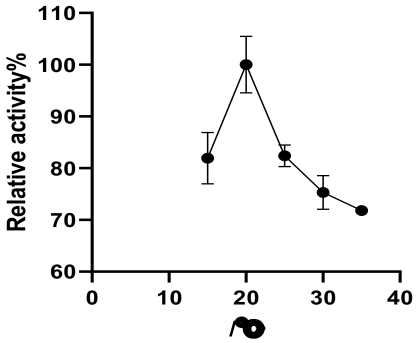


**Supplemental Figure S4** The temperature optimization of *Cs*UGT87E7. Data are presented as mean ± SD of at least three biological replicates.

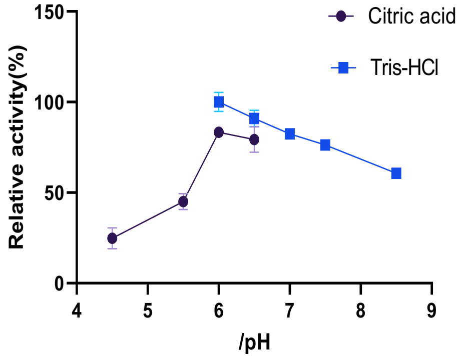


**Supplemental Figure S5** The pH optimization of *Cs*UGT87E7. Data are presented as mean ± SD of at least three biological replicates.

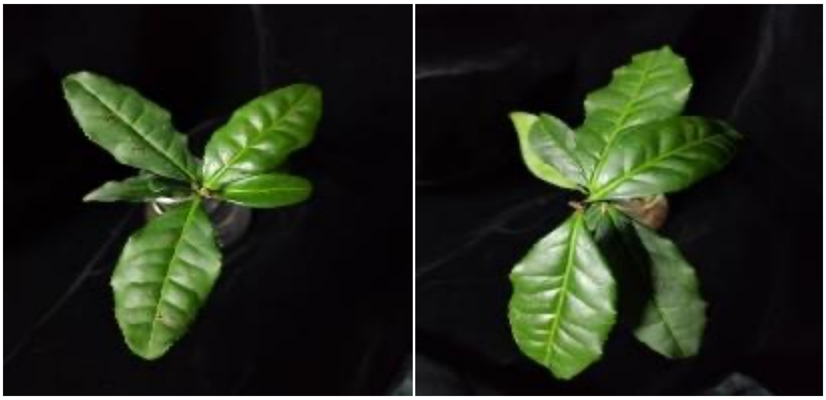

sODN AsODN

**Supplemental Figure S6** The leaf surface situation of tea plant 1day after fungus infection. AsODN, antisense oligonucleotide suppression; sODN, sense oligonucleotide
